# Construction of a *Daphnia*-sustaining microcosm using purely inorganic medium: a system designed to investigate endogenous nutrient biosynthesis

**DOI:** 10.7717/peerj.21882

**Published:** 2026-10-29

**Authors:** Kunihiko Kakazu

**Affiliations:** Naha, Okinawa, Japan

**Keywords:** Aquatic microcosm, *Daphnia*, Sediment grain size, Anaerobic environment, B-vitamin biosynthesis, Inorganic medium

## Abstract

Existing microcosms (ecosystem models) have structural limitations such as restricted element coverage in their media, exogenous vitamin supplementation, and the absence of sediment, and have not fully reproduced the biosynthesis and cycling of nutrients from inorganic substances that occurs in natural ecosystems. In the present study, an S-type microcosm (DMC: *Daphnia* Microcosm) was constructed by combining a medium containing all 31 essential elements as inorganic compounds (DMC medium) with a quartz-sand sediment of 0.3–0.6 mm grain size. No organic nutrients such as vitamins were added to the DMC medium. In a preliminary experiment comparing a no-sediment treatment (DMC medium only) and treatments with glass beads of different sizes, both the no-sediment and large-bead treatments failed to support *Daphnia* population growth and the transplanted individuals died early, whereas in the small-bead treatment dissolved oxygen within the sediment dropped below 0.1 mg L^−1^ to form an anaerobic environment, the algal community diversified to include diatoms, and *Daphnia* reproduction was observed. In the main experiment using DMC with quartz-sand sediment, *Daphnia* consistently grew from three founders to a mean peak abundance of 23.8 ± 3.2 across three successive subcultures with three replicate lines each. The growth of *Daphnia* in a purely inorganic medium devoid of vitamins is consistent with the working hypothesis that vitamins (B12, B1, B7) are biosynthesized by microorganisms in the anaerobic sediment environment and delivered to *Daphnia* through the food web. However, direct analytical verification of in-system vitamin biosynthesis was not performed in this study and remains a key task for future work. As a complement and extension of existing methods, the DMC presents a new approach to constructing an ecosystem model intended to incorporate the biosynthesis and cycling of nutrients from inorganic substances.

## Introduction

### Structural limitations of existing microcosms: from the perspective of reproducing nutrient biosynthesis and cycling

Microcosms (ecosystem models) are systems that cultivate biological communities under controlled environmental conditions to experimentally reproduce and study ecosystem processes, and they have been widely used, including for assessing the effects of chemical substances on aquatic ecosystems (Beyers & Odum, 1993). Given the limitations of single-species tests, the development of multi-species microcosms that more closely approximate natural ecosystems has been identified as an important research direction (Yang et al., 2025). However, existing microcosm methods share a common structural limitation: they do not reproduce within the system the biosynthesis and cycling of nutrients from inorganic substances, a fundamental feature of natural ecosystems. In the following, I organize this limitation from the perspectives of medium composition, sediment, and nutrient supply.

The Kurihara microcosm (Kurihara, 1978) is an S-type (stress-selected) microcosm that uses tris phosphate (TP) medium, and its high reproducibility and stability have led to its long-standing use in the assessment of chemical effects (Sugiura, 1992; Kakazu et al., 2014; Sugiura, 2010). The *Daphnia* Microcosm (DMC) of the present study also inherits this S-type microcosm construction approach. However, TP medium is composed of only 18 elements; because it lacks silicon, diatoms cannot grow and the diversity of the algal community is constrained. Furthermore, the system has shown insufficient sensitivity to chemicals that inhibit specific biochemical reactions, such as γ-BHC (lindane) and alachlor, leading to predictions that diverge substantially from those obtained in natural ecosystems (Sugiura, 1992; Kakazu, 2014). Recently, the concept of the “biogeochemical niche” has been proposed, in which each species possesses a distinct elemental composition (elementome) and coexisting species tend to develop larger differences in elemental composition in order to avoid competitive pressure (Peñuelas et al., 2019). If this hypothesis is applied to aquatic microcosms, it is conceivable that the more restricted the set of elements supplied through the medium, the fewer niche axes are available for inter-specific differentiation, potentially constraining community diversity and the range of biochemical reactions expressible within the system. However, the findings of Peñuelas et al. (2019) are based primarily on observations of terrestrial plants, and their application to aquatic microcosms remains a hypothetical extrapolation.

The Standardized Aquatic Microcosm (SAM) (US EPA, 1996) outlined in the U.S. Environmental Protection Agency guidelines, the TriCosm proposed by Riedl et al. (2018), and the closed ecological system of Taub et al. (2025) all constitute multi-species microcosms that include *Daphnia*. However, these systems use either T82MV medium (containing 11 vitamins) or B/4 medium, which is a four-fold dilution of B medium (Taub, 2009; ASTM International, 2020); in all cases vitamin B12, thiamine (B1), and biotin (B7) are exogenously supplemented. In natural aquatic ecosystems these vitamins are biosynthesized by microorganisms and supplied through the food web (Wienhausen, Bittner & Paerl, 2022); exogenous supplementation bypasses this process and may therefore lead to underestimation of the toxic effects of chemicals that inhibit vitamin-biosynthetic pathways, or render such effects difficult to detect. In addition, the TriCosm and Taub et al. systems contain no sediment, and the SAM sediment is composed mainly of limited organic detritus; none of these systems is designed to evaluate anaerobic metabolic processes or elemental cycling within an inorganic sediment matrix. A recent review of aquatic microcosm research likewise identified as a key issue that many existing microcosms are biased toward algae, rotifers, and zooplankton such as *Daphnia*, lacking sediment and microbial communities, and are therefore insufficient for assessing the ecological effects of pollutants associated with their deposition into the sediment (Yang et al., 2025).

### Keys to nutrient cycling in natural ecosystems

In natural ecosystems, organic nutrients are biosynthesized from inorganic substances by microorganisms and circulate through the food web. Sealed microbial ecosystems have been experimentally shown to cycle carbon for several months using light alone, with metabolic functions conserved even as community composition fluctuates (De Jesús Astacio et al., 2021).

Among the components of this nutrient cycle, the B-vitamin group plays a particularly important role. Vitamin B12 (cobalamin) can be biosynthesized only by prokaryotes (Grossman, 2016); many algae require B12 (155 of 306 species examined) (Croft, Warren & Smith, 2006), and zooplankton also require B12 as an essential nutrient. *Daphnia* in particular require vitamins B12, B1 (thiamine), and B7 (biotin), and deficiencies in these vitamins cause reproductive failure and growth inhibition (Keating, 1985; Elendt & Bias, 1990; Kusari et al., 2017).

In natural aquatic ecosystems, sediment serves as the principal habitat for vitamin-producing bacteria. Sugita et al. (1994) reported that in freshwater aquaculture ponds most B12-producing bacteria are anaerobes (especially *Clostridium*) and are more abundant in the sediment than in the water column. A metagenomic analysis by Lu et al. (2020) showed that fewer than 10% of soil and sediment bacteria possess B12-biosynthetic genes, but that this small fraction underpins the B12 supply of the entire system. Furthermore, the anaerobic B12-biosynthetic pathway does not require molecular oxygen, and biochemical evidence indicates that the reducing conditions of sediment interstices favor its synthesis (Balabanova et al., 2021).

Grain size plays an important role in the formation of anaerobic environments within sediment. Jackson & Weeks (2008) used 16S rRNA gene analysis to show that bacterial community structure differs across the aquatic-sediment grain-size range (63–2,000 μm). Huanca-Valenzuela et al. (2025) further reported quantitatively that, in marine particles, anaerobic metabolism is enhanced as grain size increases because internal diffusion barriers strengthen. However, when grains are too large the interstices also become too large for an anaerobic environment to develop, suggesting the existence of an optimal grain-size range for microbial colonization and anaerobic metabolism. Suzuki et al. (2013) reported that an anaerobic culture system using glass beads of 0.5 mm grain size sustained an unknown microbial community with PCB-dechlorination activity higher than that found in soil, suggesting that this grain-size range provides a suitable habitat for anaerobic bacteria.

### Aims of this study

On this background, the aim of the present study was to describe a method for constructing an S-type microcosm (DMC: *Daphnia* Microcosm) in which *Daphnia* can grow without any exogenous addition of organic nutrients such as vitamins, by combining a medium that contains all 31 essential elements as inorganic compounds (DMC medium) with a grain-size-controlled quartz-sand sediment. The intent of the DMC is to incorporate within the system the biosynthesis and cycling of nutrients by microorganisms, so as to realize an ecosystem model that more faithfully reproduces the material cycling found in natural ecosystems. The central working hypothesis of this study is that microorganisms in the anaerobic environment formed within the grain-size-controlled sediment biosynthesize B vitamins (B12, B1, B7), which are supplied to *Daphnia* through the food web, thereby sustaining the *Daphnia* population without any exogenous vitamin addition. This working hypothesis yields the testable predictions that, despite no exogenous vitamin addition, (i) B vitamins should be detectable within the system (sediment, water column, and food particles), (ii) microorganisms capable of their biosynthesis should be present in the anaerobic sediment environment, and (iii) a supply route from these sources to *Daphnia* should exist (approaches for testing these predictions are discussed in ‘Future perspectives’). The scope of the present paper is the construction and characterization of a system for testing this hypothesis in future work; direct verification of in-system vitamin biosynthesis remains a task for future work.

## Materials and Methods

### Design principles and composition of the DMC medium

DMC medium is a synthetic medium that contains all 31 elements currently recognized as essential for life (Sakurai, 2006; Schrauzer, 2002) as inorganic compounds. The medium design rests on two principles. First, no organic nutrients such as vitamins or amino acids are added from outside, so that the system must rely on microbial in-system biosynthesis—thereby reproducing the process by which nutrients are biosynthesized from inorganic substances in natural ecosystems. Second, the concentration ratios of the elements are kept within ranges close to those found in nature so as to preserve correspondence with natural ecosystems.

Specifically, the concentrations were set to 1.5 times those reported by Huheey, Keiter & Keiter (1993) for river water (Table 1). The 1.5-fold factor was adopted because river water tends to have lower element concentrations than lake water, and because dissolved concentrations decrease during cultural succession owing to biological uptake; the extra margin compensates for this consumption. Each reagent was dissolved in pure water to prepare the medium. FeSO_4_⋅7H_2_O was dissolved in Na_2_EDTA⋅2H_2_O before being added to the medium, in order to prevent precipitation of iron ions.

**Table 1:** Composition of the DMC medium and concentrations of essential elements in river water.

Reagent	DMC medium (mg/L)	Essential element	River water (mg/L)
Na_2_CO_3_	28.2	Na	9
		Cl	8
MgCl_2_	9.4	Mg	4.1
Na_2_SiO_3_⋅9H_2_O	60.7	Si	4
MgSO_4_⋅7H_2_O	38.1	S	3.7
KCl	5.9	K	2.3
CaCl_2_⋅2H_2_O	8.3	Ca	1.5
		C	1.2
FeSO_4_⋅7H_2_O	5	Fe	0.67
Na_2_EDTA⋅2H_2_O	2.73		
NaNO_3_	1.02	N	0.25
KF	0.46	F	0.1
SrCl_2_⋅6H_2_O	0.23	Sr	0.05
KH_2_PO_4_	0.13	P	0.02
NaBr	0.04	Br	0.02
H_3_BO_3_	0.09	B	0.01
ZnSO_4_⋅7H_2_O	0.07	Zn	0.01
MnCl_2_⋅4H_2_O	0.027	Mn	0.005
CuSO_4_⋅5H_2_O	0.029	Cu	0.005
KI	0.01	I	0.005
PbCl_2_	0.006	Pb	0.003
NH_4_VO_3_	0.003	V	0.001
CrCl_3_⋅6H_2_O	0.008	Cr	0.001
Na_2_HAsO_4_⋅7H_2_O	0.006	As	0.001
RbCl	0.002	Rb	0.001
Na_2_MoO_4_⋅2H_2_O	0.004	Mo	0.001
NiCl_2_⋅6H_2_O	0.0018	Ni	0.0003
Co(NO_3_)_2_⋅6H_2_O	0.0015	Co	0.0002
Na_2_SeO_4_	0.0007	Se	0.0002
SnCl_2_⋅2H_2_O	0.00011	Sn	0.00004
LiCl	0.027	Li	0.003
AgCl	0.0006	Ag	0.0003

Reagent concentrations were set so that each element corresponds to 1.5 times its river-water concentration. Element concentrations are based on Huheey, Keiter & Keiter (1993).

### Sediment design

The sediment was designed to diversify the habitable space for microorganisms within the microcosm and to promote the formation of an anaerobic environment.

In the preliminary experiment, to examine the necessity of sediment and the effect of grain size, a no-sediment treatment (DMC medium only, without sediment) was compared with two glass-bead (GB) treatments using BZ-1 and BZ-2 beads (AS ONE, Osaka): large-grain glass beads (L-GB; diameter 1.5–2.5 mm, 200 g) and small-grain glass beads (S-GB; diameter 0.991–1.397 mm, 200 g).

In the main experiment, quartz sand was adopted as the sediment material for the following reasons. First, light penetrates to the bottom of glass-bead sediment, producing an environment that differs from natural sediment. Second, quartz sand is a standard material used in the OECD sediment toxicity test (OECD, 2010) and provides good reproducibility across tests. The quartz sand used (Albany 27; SiO_2_ 99.8%; 87% of grains within 0.3–0.6 mm; Tochu, Aichi, Japan) was loaded at 275 g per culture vessel.

### Collection of microorganisms and successive subculturing

This study adopted the S-type (stress-selected) microcosm approach. In an S-type microcosm, natural communities collected from multiple locations are mixed and propagated through repeated successive subculturing, allowing an environment-adapted community to stabilize; the resulting species composition is largely free of arbitrary human selection and exhibits high reproducibility (Kurihara, 1978).

The microbial constituents of the DMC were initially established by collecting microorganisms with plankton nets from lakes and rivers across eastern Japan (the Abukuma River, Lake Kasumigaura, and others) and inoculating them into culture vessels containing DMC medium and sediment. Subsequent successive subcultures were performed while removing impurities such as soil particles, and the biological succession was observed. The main experiment was initiated only after more than one year and at least eight successive transfers from the initial community collection, once it had been confirmed that the system had stabilized under conditions with no exogenous vitamin addition. *Daphnia* were collected from a pond near Fukushima University (37°41′12.4″N, 140°27′17.1″E); the clonal lineage of *Daphnia* sp. used was morphologically similar to the *D. pulex* group (Fig. 1). Molecular identification was not performed, so the species-level identity remains unconfirmed.

**Figure 1:**
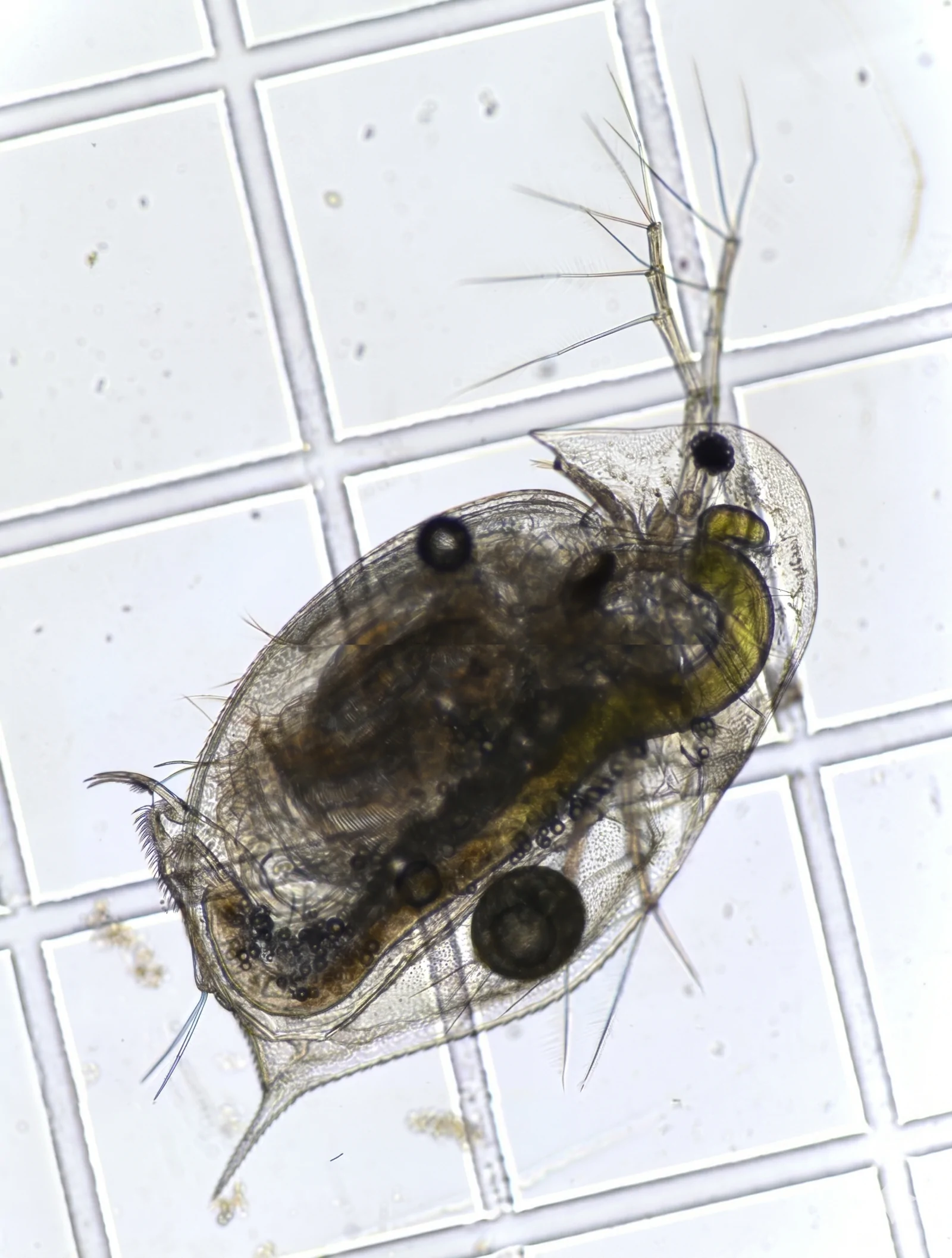
Photograph of the *Daphnia* sp. clonal lineage used in this study. The morphology was similar to that of the *D. pulex* group. Individuals were collected from a pond near Fukushima University. The background grid is a slide glass with 1-mm grid spacing, providing a length reference. Photo credit: Kunihiko Kakazu.

### Culture conditions

Sediment and 700 mL of DMC medium were placed into a 1,000-mL Erlenmeyer flask, the flask was closed with a silicone stopper, and the assembly was autoclaved (121 °C, 20 min). After cooling, 30 mL of culture solution containing sediment-interior microorganisms was inoculated from an ongoing DMC subculture. Cultures were maintained at 20 °C and an illuminance of 2,000 lx (14.5-h light/9.5-h dark cycle) under stationary conditions. A fluorescent lamp (FL20SS-W/18, National) was used as the light source. The temperature was selected based on the *Daphnia* culture temperature recommended by the OECD (18–22 °C) (OECD, 2012), and the light/dark cycle was set with reference to the mean daylight duration on the summer solstice in Japan. An overview of the assembled DMC is shown in Fig. 2.

**Figure 2:**
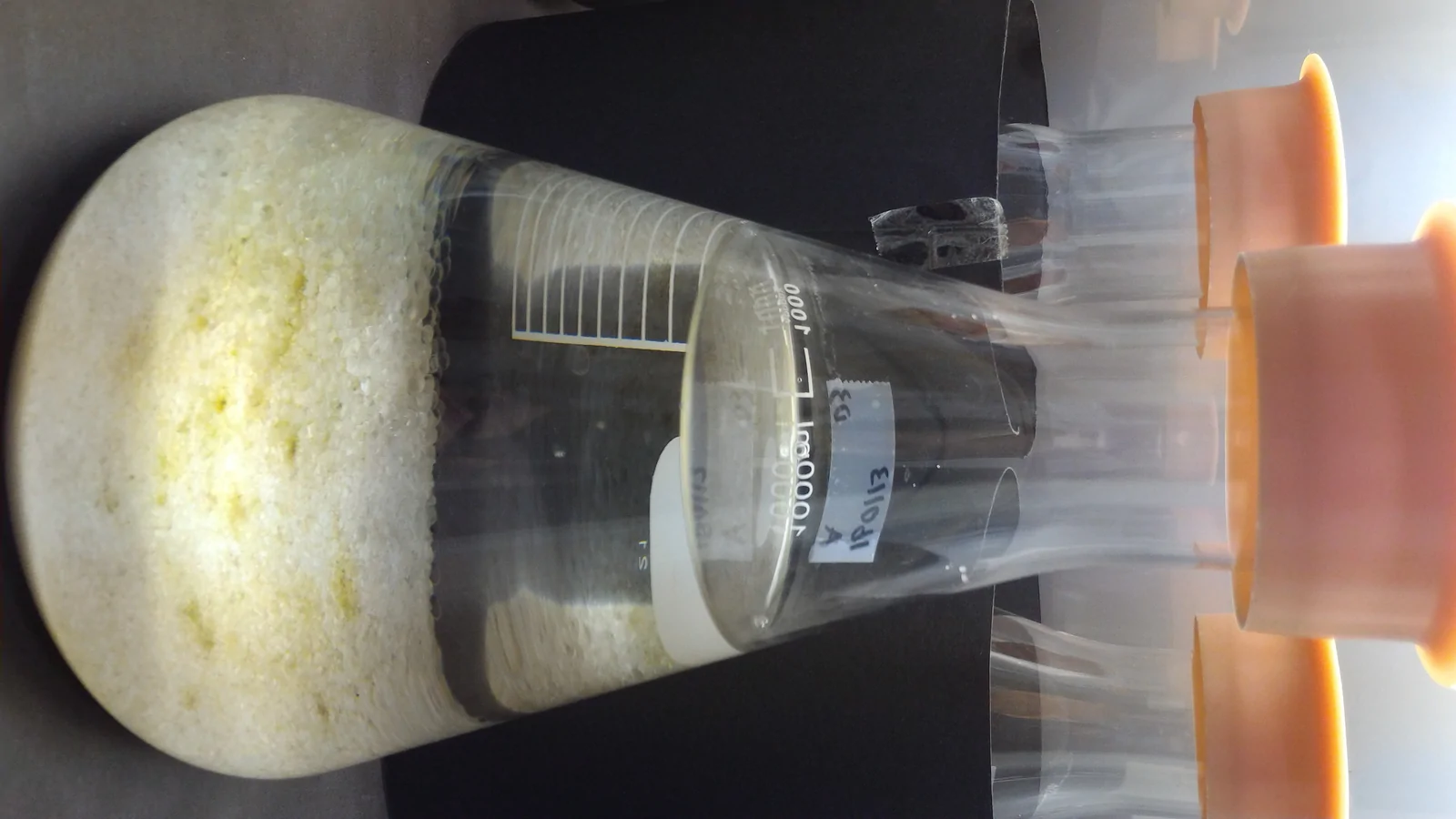
Overview of the assembled DMC. A 1,000-mL Erlenmeyer flask contains 275 g of quartz sand (Albany 27, grain size 0.3–0.6 mm) as sediment and 700 mL of DMC medium, sealed with a silicone stopper. Photo credit: Kunihiko Kakazu.

Fourteen days after the start of culture, once stabilization of the microcosm had been confirmed, three *Daphnia* individuals without eggs were transplanted into each flask. Three replicate lines were prepared for each subculture, and a total of three successive subcultures (Subculture I–III) were performed. At the end of each subculture, the sediment of each line was disturbed and a culture-solution sample was collected; a pooled inoculum consisting of 10 mL from each of the three previous lines (30 mL in total) was then transferred to each of the new flasks (three lines) of the next subculture, containing sterilized fresh DMC medium (700 mL) and sediment. After a 14-day stabilization period following inoculation, three *Daphnia* individuals without eggs were transplanted into each line. The three successive subcultures (Subculture I–III) in this experiment are therefore not parallel replicates but a sequential series that inherits the microbial community, with each new line restarting as a pooled community drawn from all three previous lines, not as a one-to-one lineage.

### Measurements

Dissolved oxygen (DO) and temperature of the culture solution were measured continuously with a fluorescence-based DO meter (ProODO, YSI). *Daphnia* individuals were counted visually every two days. The main biotic constituents of the DMC were observed and photographed by light microscopy; representative photomicrographs are provided in Fig. S1.

### Data analysis

For each time point, the mean and standard deviation (SD) of the three replicate lines were calculated for *Daphnia* abundance. For each subculture, the peak abundance of each line (the maximum during the observation period) was identified, and peak abundances were compared across subcultures as mean ± SD. As an index of reproducibility, the coefficient of variation (CV = SD/mean × 100%) was calculated at each sampling timepoint. To evaluate reproducibility at each phase of population dynamics, three phases were defined for each subculture relative to its peak value (the maximum of the three-replicate mean time series): growth phase (mean abundance below 90% of the peak value and before the peak was reached), peak phase (mean abundance at or above 90% of the peak), and decline phase (the period of decrease following the peak phase). The range of time-resolved CVs across the timepoints within each phase was tabulated. The calculated CVs were compared with values reported in existing aquatic micro/mesocosm studies (Kraufvelin, 1998; Sanderson, 2002).

Subculture I measurements were terminated for inoculation purposes on day 22 post-transplantation (day 36 of culture); measurements were continued until day 40 post-transplantation for Subculture II and until day 32 post-transplantation for Subculture III. Owing to the small sample size (*n* = 3), statistical testing was not performed and only descriptive statistics are reported.

### Ethics statement

This study involved only invertebrate animals (*Daphnia* sp.; Crustacea: Cladocera) and environmental microbial communities collected from public freshwater bodies in Japan. No vertebrate animals, cephalopods, or human participants were involved. Under the applicable Japanese national legislation and institutional regulations, research on invertebrates does not require review or approval by an animal ethics committee.

## Results

### Preliminary experiment: effects of sediment presence and grain size

To examine the necessity of sediment and the effect of grain size, a preliminary experiment was conducted comparing a no-sediment treatment (DMC medium only) with two glass-bead treatments (L-GB: diameter 1.5–2.5 mm; S-GB: diameter 0.991–1.397 mm).

Over the course of system development, the no-sediment treatment (DMC medium only) and the L-GB treatment repeatedly showed, on multiple occasions, the same tendency: green algae and cyanobacteria dominated and the number of algal species was limited, and the transplanted *Daphnia* produced no neonates and died before population growth could be established. In contrast, switching the sediment to S-GB caused pennate diatoms to newly appear (morphologically similar to the forms observed in the main experiment; cf. Figs. S1C–S1E), increasing the species diversity of the algal community, and *Daphnia* reproduction (neonate production) and an increase in abundance were recorded. These differences in the biotic community associated with the sediment condition were reproducibly observed throughout system development and motivated the anaerobic-sediment design adopted in this study.

Dissolved oxygen within the sediment was measured over a 41-hour period: in the L-GB treatment, DO remained at approximately 1.0–1.7 mg L^−1^ (mean 1.2 mg L^−1^) throughout the measurement period, whereas in the S-GB treatment DO declined rapidly with time, falling below 0.1 mg L^−1^ within approximately 15 h and remaining at this level thereafter (Fig. 3). These results confirm the formation of an anaerobic environment in the interior of small-grain sediment.

**Figure 3:**
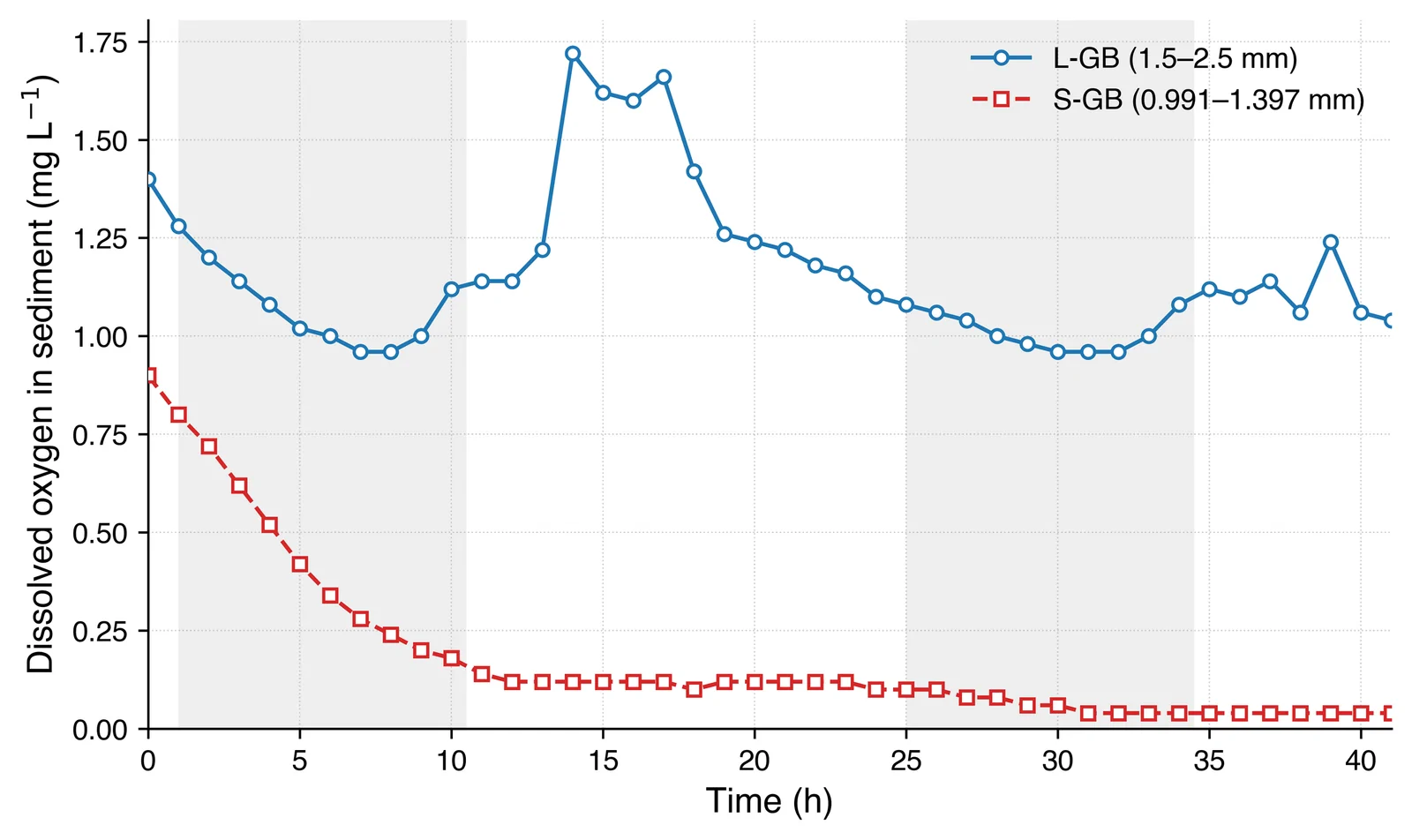
Time course of dissolved oxygen (DO) within the glass-bead sediments of two grain sizes during the preliminary experiment. L-GB: large glass beads (diameter 1.5–2.5 mm); S-GB: small glass beads (diameter 0.991–1.397 mm). DO was monitored for 41 h with a fluorescence-based DO meter in a single preliminary measurement for each treatment.

### Main experiment: stability of the DMC

For the DMC with quartz-sand sediment (0.3–0.6 mm grain size), the dissolved oxygen concentration of the culture solution was continuously monitored for 1,488 h (62 days) from the start of culture (Fig. 4). During the early stage of culture, oxygen production by photosynthesis was lower than respiratory consumption, and DO decreased. From about day 5 onwards, however, photosynthesis began to increase and DO turned upward. After day 14, a stable diel pattern of oxygen production during the light phase and consumption during the dark phase was established, indicating that the in-system ecosystem had reached a steady state.

**Figure 4:**
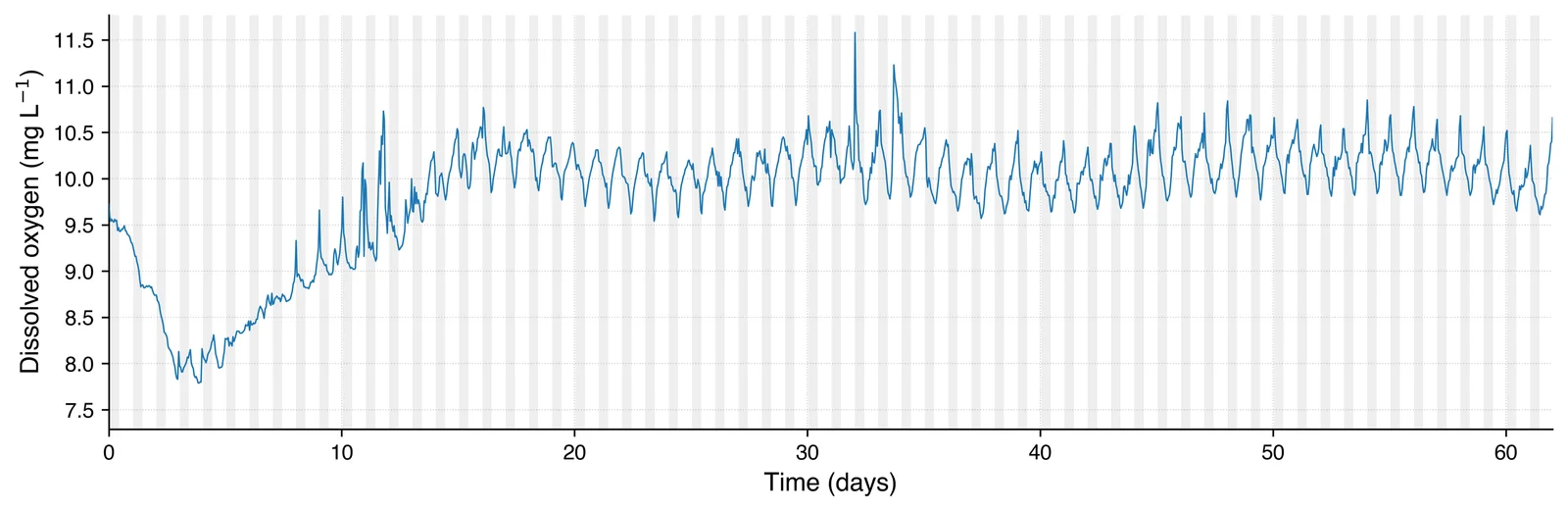
Time course of dissolved oxygen (DO) in the culture solution of the DMC with quartz-sand sediment (0.3–0.6 mm grain size) during the main experiment. DO was continuously monitored for 1,488 h (62 days) from the start of culture. A stable diel pattern of photosynthetic production during the light phase and respiratory consumption during the dark phase was established after day 14.

The temperature of the culture solution fluctuated around 20.7 °C, rising slightly during the light phase and decreasing during the dark phase (Fig. 5). During the early period (about days 0–33), the diel temperature amplitude was somewhat larger (19.7–21.3 °C); from the mid-period onwards (after day 34) the amplitude narrowed (20.4–20.9 °C).

**Figure 5:**
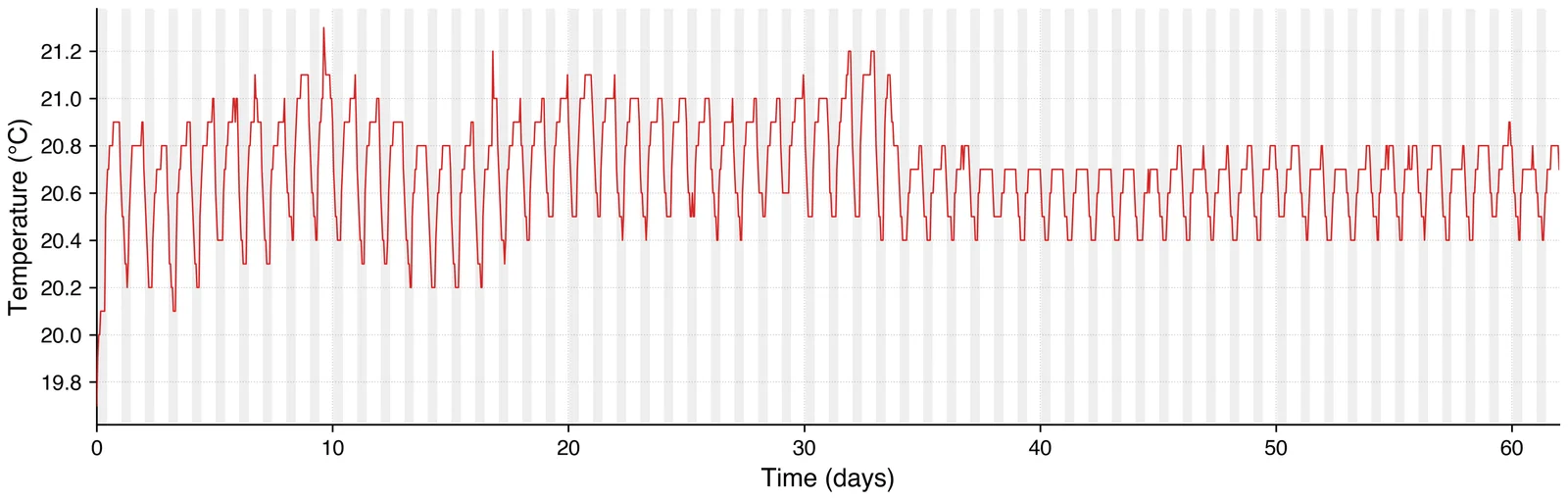
Time course of culture-solution temperature in the DMC during the main experiment. Temperature fluctuated around 20.7 °C, rising during the light phase and decreasing during the dark phase. The diel amplitude was larger during the early period (days 0–33; 19.7–21.3 °C) and narrowed from day 34 onwards (20.4–20.9 °C).

### Main experiment: *Daphnia* growth

On day 14 of each subculture, when the DMC was in its stable phase, three *Daphnia* individuals were transplanted, and changes in abundance were measured across three sequential subcultures that inherit the microbial community (Subculture I–III). Means and standard deviations (SD) of the three lines at each time point are shown in Fig. 6.

**Figure 6:**
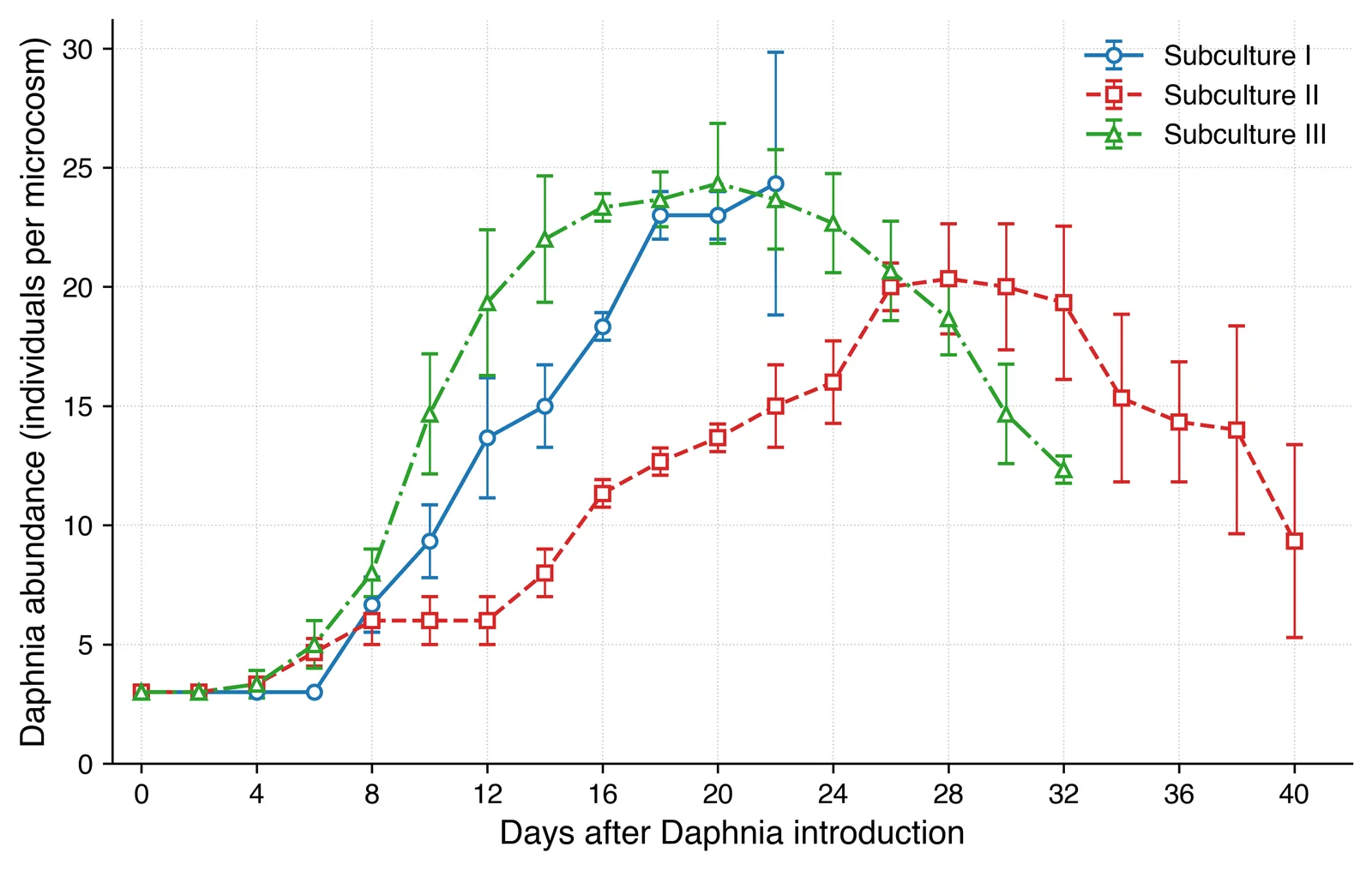
*Daphnia* abundance dynamics across the three successive subcultures (Subculture I–III) of the DMC. Values are mean ± SD of three replicate lines (*n* = 3) at each time point. Three *Daphnia* individuals without eggs were transplanted on day 14 of each subculture (day 0 post-transplantation in the time axis used here). Subculture I was measured until day 22 post-transplantation, Subculture II until day 40, and Subculture III until day 32.

In all subcultures, *Daphnia* began to proliferate 4–8 days after transplantation and reached a peak. In Subculture II and III, a subsequent decline phase was also observed, whereas Subculture I was still on an increasing trend at the end of the observation period (day 22 post-transplantation/day 36 of culture) (Fig. 6).

**Subculture I.** A marked increase in abundance began on day 8 post-transplantation (day 22 of culture), with the peak reached on days 18–22 post-transplantation. The mean abundance on the final day of measurement (day 22 post-transplantation) was 24.3 ± 5.5.

**Subculture II.** The growth rate was slower than in Subculture I; the peak (mean 20.3 ± 2.3) was reached on days 26–28 post-transplantation (days 40–42 of culture). Abundance then declined, falling to 9.3 ± 4.0 by day 40 post-transplantation. A possible cascade is that grazing pressure by *Daphnia* depleted the algal community, and the resulting decline in algae-mediated vitamin supply led to the observed population decrease.

**Subculture III.** Growth began 4 days after transplantation, showing the fastest growth among the three subcultures. The peak (mean 24.3 ± 2.5 on day 20 post-transplantation) was reached on days 16–20 post-transplantation (days 30–34 of culture), followed by a decline.

Subculture I was measured until day 22 post-transplantation (day 36 of culture), Subculture II until day 40 post-transplantation, and Subculture III until day 32 post-transplantation. Detailed time-resolved data are provided in Table S1.

Across the three successive subcultures, the across-subculture mean of the peak abundance was 23.8 ± 3.2, and a stable 7- to 9-fold increase from the initial three founders was consistently reproduced. The number of days to peak varied across subcultures, with Subculture II showing a delay relative to the others (Table 2). The time-resolved CVs ranged 3–20% in the growth phase, 3–23% in the peak phase, and 5–43% in the decline phase. Peak-phase CVs were low in Subculture III (3–12%) and Subculture II (5–17%), while Subculture I showed a somewhat wider range (4–23%). The maximum CV of 43%, observed at the end of the decline phase of Subculture II (day 40 post-transplantation / day 54 of culture, 9.3 ± 4.0 individuals), reflects the inherent inflation of CV as the mean abundance dropped to low values.

**Table 2:** Summary of population dynamics and phase-specific coefficients of variation (CV) for each subculture.

Subculture	Peak abundance^1^	Days to peak^2^	Growth-phase CV^3^	Peak-phase CV^3^	Decline-phase CV^3^
Subculture I	25.7 ± 3.2	20.7 ± 2.3	3–18% (5 pts)	4–23% (3 pts)	not measured
Subculture II	20.7 ± 2.1	26.7 ± 1.2	4–18% (11 pts)	5–17% (4 pts)	17–43% (4 pts)
Subculture III	25.0 ± 2.0	18.0 ± 2.0	13–20% (5 pts)	3–12% (6 pts)	5–14% (4 pts)
Across-subculture mean	23.8 ± 3.2	—	—	—	—

**Notes.**

1 Mean ± SD of the per-line peak abundance—*i.e.*, the maximum reached by each of the three lines during the observation period, averaged across lines (*n* = 3; individuals per 700 mL). Because the three lines peaked on slightly different days, this value is generally equal to or slightly higher than the peak of the three-line mean time series quoted in ‘Main experiment: *Daphnia* growth’. Per-line peak values are provided in Table S1.

2 Days after *Daphnia* transplantation (*n* = 3, mean ± SD).

3 Range of time-resolved CVs (= SD/mean × 100%) at the timepoints within each phase. The numbers in parentheses are the numbers of timepoints. Phase definitions: growth phase = the post-transplantation increase period during which the mean abundance at each timepoint was below 90% of the peak value of that subculture; peak phase = the period in which the mean abundance remained at or above 90% of the peak; decline phase = the decreasing period following the peak phase. Subculture I did not enter the decline phase within its observation period.

## Discussion

### Implications of *Daphnia* growth in an inorganic medium

Despite the DMC medium containing no organic nutrients such as vitamins, *Daphnia* grew consistently across three successive subcultures (mean peak abundance 23.8 ± 3.2 across all subcultures). Deficiencies of B12, B1, or B7 are each known to cause reproductive failure and growth inhibition in *Daphnia*, which therefore requires an external supply of these vitamins for development and reproduction (Keating, 1985; Elendt & Bias, 1990; Kusari et al., 2017). The fact that *Daphnia* reproduced within the DMC, which contains no added vitamins, is therefore consistent with the working hypothesis that these vitamins were biosynthesized in-system by microorganisms and supplied to *Daphnia* through the food web.

For this working hypothesis to hold, alternative routes other than in-system biosynthesis—that is, carry-over from outside the system—must be excluded. Two alternative hypotheses are considered: (i) residual vitamins carried over from the initial collection of the natural community or from the *Daphnia* transplantation, and (ii) supply from internal reserves within the transplanted parental *Daphnia* individuals.

For the first hypothesis (residual carry-over from outside), the DMC system had been propagated over more than one year and at least eight successive subcultures before the start of the present experiment, and at each transfer the ratio of inoculum (30 mL) to fresh medium (700 mL) was 30:700; because the inoculum is added to the fresh medium, the total culture volume is 730 mL, giving a residual fraction per subculture of 30/730 ≈ 0.041 (approximately 4.1%). Even if vitamins at concentrations comparable to those of a representative standard medium (M4 medium: B12 = 1 µg L^−1^, B1 = 75 µg L^−1^, B7 = 0.75 µg L^−1^) (Elendt & Bias, 1990) had been introduced at the initial collection of the natural community or in the preparation of the zeroth generation, the residual concentration after three subcultures would be 0.041^3^ ≈ 6 × 10^−5^ of the original, and after eight subcultures it would be approximately 8.1 × 10^−12^—effectively zero. This level is more than ten orders of magnitude below the B12 threshold for reproductive impairment in *D. pulex* (∼375 ng L^−1^) reported by Keating (1985); the population growth observed in the present experiment is therefore quantitatively untenable as an outcome of residual carry-over of initially introduced vitamins.

For the second hypothesis (parental reserves), direct measurements in other zooplankton (copepods) indicate that the thiamine content per individual varies with genus, sea area, and season, ranging broadly from approximately 0.2–2.5 pmol per individual (about 0.05–0.7 ng; estimated from Fig. 1C of Fridolfsson et al. (2019). Assuming *Daphnia* values to be of the same order of magnitude and using the conservative upper bound, the B1 carried over from the three transplanted parental individuals would amount to at most approximately 2 ng; distributed in 700 mL of culture solution this would correspond to about 3 ng L^−1^, less than 10^−4^ of the B1 concentration in M4 medium (75 µg L^−1^). Furthermore, thiamine is known to undergo relatively rapid turnover within cells (the turnover time of ThDP in cultured mouse neuroblastoma cells, neuro-2a, is about 17 h) (Bettendorff, 1994). These quantitative constraints indicate that parental reserves alone cannot meet the demand of the newly proliferated population (an approximately 8-fold increase, from 3 to a mean of 23.8 individuals). This conclusion is also consistent with the control data from the preliminary experiment: in the no-sediment treatment (DMC medium only) and the large-bead treatment (L-GB), which used the same parental *Daphnia* individuals, no in-system anaerobic locus for vitamin biosynthesis was established, and the transplanted individuals died early without producing neonates (‘Preliminary experiment: effects of sediment presence and grain size’). If parental reserves alone were sufficient to sustain reproduction, at least some growth or neonate production would have been expected under these conditions. These control-like observations, repeatedly made throughout system development, are inconsistent with the parental-reserve hypothesis.

Since these alternative hypotheses are excluded on both quantitative and experimental grounds, the sustained growth of *Daphnia* observed in the present study is most parsimoniously explained by a dependence on vitamins synthesized within the system. This nonetheless remains a working hypothesis supported by circumstantial evidence rather than by direct vitamin quantification (‘Limitations’); direct verification that in-system vitamin biosynthesis is indeed responsible for sustaining the *Daphnia* population remains a task for future work, and approaches for this verification are described in ‘Future perspectives’. The most likely source of vitamin production is the anaerobic bacterial community within the sediment. B12 can be biosynthesized only by prokaryotes (Grossman, 2016), and anaerobic bacteria such as *Clostridium* have been reported to produce substantial amounts of B12 in sediment (Sugita et al., 1994). Both anaerobic (early cobalt-insertion) and aerobic pathways for B12 biosynthesis exist, but the anaerobic pathway does not require molecular oxygen, and biochemical evidence indicates that the reducing conditions within sediment interstices favor it (Balabanova et al., 2021). The metagenomic analysis of Lu et al. (2020) showed that, although fewer than 10% of soil and sediment bacteria carry B12-biosynthetic genes, this minority community can sustain the B12 supply of the entire system.

The possibility that *Daphnia*-associated bacteria also contribute to vitamin biosynthesis cannot, however, be ruled out. Cooper & Cressler (2020) reported that the genomes of *Daphnia*-associated bacteria encode complete biosynthetic pathways for B12 and B7. That finding, however, was obtained under rearing conditions using COMBO medium (which contains vitamins) and does not constitute direct evidence for functional vitamin supply by these bacteria. Disentangling the respective contributions of sediment anaerobic bacteria and *Daphnia*-associated bacteria is an important future research task.

In natural ecosystems, distinct taxonomic groups have been reported to specialize in the biosynthesis of different vitamins, exhibiting “mosaic patterns” (Gómez-Consarnau et al., 2018); about one-third of particle-attached bacteria show B-vitamin auxotrophy, and dynamics in which producers support these auxotrophs have been observed (Gregor et al., 2025). For algae as well, more than half of the 306 species surveyed are auxotrophic for B12 and 22% for B1, depending on external supply (Croft, Warren & Smith, 2006). It is therefore conceivable that, within the DMC, a “vitamin-producing bacteria → vitamin-auxotrophic bacteria → algae → *Daphnia*” supply cascade emerges as a community-level division of roles. Moreover, thiamine (B1) is poorly transferred across the food web: approximately 59% is lost as it moves from phytoplankton to zooplankton, and zooplankton retain only about 41% of phytoplankton thiamine (Hylander et al., 2024, Table 1). Stable B1 supply to *Daphnia* therefore requires substantial primary production within the sediment, underscoring the importance of the sediment functioning as a “biochemical reactor”.

Beyond *Daphnia*, light microscopy of the DMC also revealed the co-occurrence of multiple biological groups, including filamentous and colonial green algae, pennate diatoms, heliozoan protozoa, and small freshwater annelids of the genus *Aeolosoma* (Aeolosomatidae) (Fig. S1). The simultaneous occurrence of taxa spanning prokaryotes, eukaryotic algae, protozoa, and metazoa within a single inorganic-medium system is consistent with the view that a multi-trophic community had become established.

### Anaerobic environment and bacterial community formation as a function of sediment grain size

In the preliminary experiment, dissolved oxygen within L-GB sediment (diameter 1.5–2.5 mm) was approximately 1.2 mg L^−1^, whereas in S-GB sediment (diameter 0.991–1.397 mm) it dropped below 0.1 mg L^−1^, forming a clearly anaerobic environment. This grain-size-dependent formation of an anaerobic environment is considered to be the key that enabled algal diversification and *Daphnia* growth. Sediment grain size governs both the formation of anaerobic conditions and the establishment of bacterial communities, and that the 0.3–0.6 mm quartz sand adopted for the DMC lies in a grain-size band satisfying both requirements is supported by three independent lines of prior research.

First, without grain sizes above a certain threshold, no significant diffusion barrier develops within or between particles, and anaerobic metabolism is unlikely to develop. Huanca-Valenzuela et al. (2025) used 16S rRNA absolute quantification across seven size fractions of marine suspended particles (0.2 to > 500 μm), and showed that bacterial density rose markedly only in the fractions of 180 µm and larger at depth (especially in the oxygen-minimum zone), with communities involved in anammox and anaerobic nitrogen cycling becoming dominant; the <53 µm fraction showed no such increase. Direct application to freshwater sediment requires caution, but the physical principle that microniche formation requires a certain grain-size scale is shared; if the DMC quartz sand were below 0.1 mm, anaerobic community development would likely be similarly suppressed.

Second, grain size determines not only the strength of the anaerobic environment but also the composition of the bacterial community that establishes itself. Jackson & Weeks (Jackson & Weeks, 2008), using 16S rRNA analysis of streambed sediments, reported that the communities separated into three clusters of 63–125 µm, 125–1,000 µm, and 1,000–2,000 µm; ribotypes belonging to the Verrucomicrobia–Planctomycetes lineage, which are closely associated with anaerobic and microaerobic environments, accounted for 20–30% of all ribotypes in the 63–1,000 µm fractions but dropped below 4% in the coarse 1,000–2,000 µm fraction. The 0.3–0.6 mm quartz sand of the DMC falls within the intermediate cluster, while L-GB (1.5–2.5 mm) corresponds to the coarse cluster.

Third, that grain sizes simultaneously satisfying the two conditions above also sustain functional metabolic activity has been shown in an independent culture experiment. Suzuki et al. (2013) reported that in a glass-bead anaerobic culture system, PCB-dechlorination activity exceeding that of soil was obtained only at 0.5 mm grain size, while no comparable activity was observed at 0.05, 1.0, or 2.0 mm. Inhibition of mass transfer by interstitial blockage is anticipated at very small grain sizes, while at very large grain sizes the diffusion barrier is lost; this pattern is also consistent with the success of the S-GB treatment and the failure of the L-GB treatment in the present study.

These three independent findings collectively suggest that the grain-size range around 0.3–0.6 mm constitutes a “habitat-compatible window” simultaneously satisfying three conditions: (i) formation of anaerobic microniches within the interstices, (ii) colonization by anaerobic and microaerobic communities, and (iii) compatibility of mass transfer with metabolic activity. The quartz-sand grain size adopted in the DMC falls within this window, and the observed stabilization of the system and the sustained growth of *Daphnia* may be interpreted as the result of this grain-size choice potentially providing the substrate for the expression of anaerobic metabolic functions, including in-sediment vitamin biosynthesis.

It should be noted that the DO profile within the quartz-sand (0.3–0.6 mm) sediment used in the main experiment was not directly measured, and an extrapolation from the glass-bead preliminary data of Fig. 3 is therefore required. However, in packed sediment, decreasing grain size reduces interstitial diameter and the effective diffusion coefficient, while the increase in specific surface area enhances per-unit-volume oxygen consumption; both effects act in the direction of strengthening the anaerobic conditions. In the preliminary experiment, a steep decline in DO was observed as grain size decreased from L-GB (1.5–2.5 mm; about 1.2 mg L^−1^) to S-GB (0.991–1.397 mm; <0.1 mg L^−1^) (Fig. 3), and it is physically predicted that the still finer quartz sand (0.3–0.6 mm) used in the main experiment would form at least as strong an anaerobic environment within the sediment.

### Positioning of the DMC: comparison with existing methods

The DMC is clearly distinguished from existing microcosm methods by the following three features.

First, in terms of medium composition. DMC medium contains all 31 essential elements as inorganic compounds, and no organic nutrients including vitamins are added from outside. By contrast, the TP medium of the Kurihara microcosm is restricted to 18 elements, and the T82MV medium of the SAM supplements 11 vitamins externally. This DMC-medium approach makes it possible to incorporate within the system the fundamental natural-ecosystem process of nutrient biosynthesis from inorganic substances. The comprehensive provision of multiple elements may have provided the substrate for the coexistence of microbial species with differing elemental requirements, potentially underpinning the expression of diverse metabolic functions, including vitamin biosynthesis.

Second, in terms of sediment design. The DMC uses a grain-size-controlled quartz sand (0.3–0.6 mm) as its sediment, deliberately designed to form an anaerobic environment and to diversify the microbial habitat. The TriCosm (Riedl et al., 2018) includes no sediment, and the Kurihara microcosm likewise does not use sediment. OECD TG 218/219 employs quartz sand but in single-species tests that do not provide ecosystem-level assessment.

Third, in terms of construction principle. The DMC employs the S-type microcosm approach, in which microbial communities collected from natural environments are stabilized through successive subcultures. The species composition within the system is thereby determined not by arbitrary human selection but by natural selection under the given environmental conditions.

Importantly, the design intent of the DMC is not the “negation” of existing methods but their “complement and extension”. Upon the well-established toxicity-testing framework of the Kurihara microcosm, the DMC seeks to add comprehensive element coverage and a sediment-based anaerobic environment, broadening the range of chemical effects that can be detected. Through the construction of the DMC, the evaluation of ecotoxicological endpoints that are difficult to detect with existing methods—such as effects on vitamin-biosynthetic pathways and on sediment-mediated material cycling—is expected to become feasible in the future. Yang et al. (2025) identified the development of multi-species microcosms encompassing sediment and microbial communities, beyond zooplankton-centric existing systems, as a future priority of aquatic-microcosm research, and the DMC may be regarded as one implementation in line with this direction.

The results of this study, when viewed in light of quantitative reproducibility indices in microcosm research, are placed within the range reported in the existing literature. Kraufvelin (1998) characterized a coefficient of variation (CV) of 20–30% as a practically acceptable range for micro/mesocosm experiments. According to a review by Sanderson (2002) of pesticide micro/mesocosm studies over the preceding 20 years, the mean reported CV was 45% (32% for small-scale indoor systems) with an average of 3–4 replicates, and animal-endpoint variables tended to show greater variability than plant or abiotic variables. In the present study, CVs were calculated at each measurement timepoint along the time series and aggregated by phase (growth, peak, decline) of population dynamics (Table 2). The across-subculture CV of the peak abundance was 13.4% (23.8 ± 3.2; n = 3  × 3 subcultures), falling below the lower bound of the practical 20–30% range of Kraufvelin (1998). The peak-phase, time-resolved CVs were 3–12% in Subculture III, 5–17% in Subculture II, and 4–23% in Subculture I, falling below the lower bound of the practical range of Kraufvelin (1998) at every timepoint except Day 36 of Subculture I (23%), and falling below the mean CV of small-scale indoor systems reported by Sanderson (2002) (32%) at all timepoints. Growth-phase CVs were also within 3–20%, again within the same standard. The replicate count of *n* = 3 likewise matches the field average (3–4) reported by Sanderson (2002). The maximum 43% CV observed at the end of the decline phase (Subculture II, Day 54) reflects the inherent inflation of CV as the mean dropped to 9.3 individuals and represents a different phase of the dynamics, not the reproducibility of the central observational endpoint. It should be noted that the three replicate lines within each subculture were established from a common pooled inoculum drawn from the stabilized community. The CVs reported here therefore quantify the reproducibility with which the same ecosystem is reconstructed from a shared source community—the property of primary relevance for a microcosm intended for repeated, standardized testing—rather than independent biological replication across source communities. These results indicate that the reproducibility of the DMC, constructed under the condition of no exogenous nutrient input, reaches the standard level of the existing aquatic-microcosm literature.

### Limitations

This study has several important limitations.

First, direct quantitative analysis of the vitamins (B12, B1, B7) was not performed. Although the fact that *Daphnia* grew in the DMC is consistent with in-system vitamin biosynthesis, the presence and concentrations of these vitamins were not directly confirmed by analytical methods such as HPLC, and vitamin biosynthesis therefore remains an inference.

Second, microbial community analysis was not performed. Because neither 16S rRNA gene analysis nor metagenomic analysis was conducted, the identities of the microorganisms present in the DMC sediment and water column, and which of them carry vitamin-biosynthetic genes, remain unknown. The respective contributions of sediment anaerobic bacteria and *Daphnia*-associated bacteria have likewise not been resolved.

Third, comprehensive analyses of algal biomass and species composition, and quantification of vitamin concentrations, were not performed. The interpretation in ‘Main experiment: *Daphnia* growth’ that the population decline resulted from an “algal-depletion-to-vitamin-supply-collapse” cascade therefore rests on circumstantial evidence; direct verification through time-resolved quantification of the algal community and vitamin concentrations remains a future task.

Fourth, the rationale for setting DMC-medium elemental concentrations at 1.5 times those of river water is not fully quantitative. A systematic examination of optimal elemental concentrations will be required in future work.

Fifth, the optimal sediment grain-size range was inferred from the glass-bead preliminary experiment together with prior literature. A grain-size series using quartz sand itself (*e.g.*, a sub- 0.1 mm fine sand and a coarse sand alongside the 0.3–0.6 mm sand) was not run, so direct confirmation that 0.3–0.6 mm is optimal for the DMC awaits a quartz-sand grain-size series.

Sixth, statistical limitations and constraints on initial-condition standardization apply. The number of replicates per subculture was *n* = 3, comparable to the field average (3–4) of micro/mesocosm studies reviewed by Sanderson (2002); as noted in that review, however, this sample size affords limited statistical power. The present study reports only descriptive statistics (mean ± SD) and does not evaluate the statistical significance of between-subculture differences. The slight reduction in peak abundance in Subculture II (20.7 ± 2.1) and the delay in reaching the peak relative to other subcultures (26.7 ± 1.2 days) can be interpreted within the known tendency for animal-based endpoints to show higher variability than plant and abiotic endpoints (Sanderson, 2002); claiming complete inter-subculture reproducibility, however, would require verification using larger sample sizes and a greater number of subcultures. In addition, owing to constraints inherent in this small-scale experiment, the only criterion adopted for transplanted *Daphnia* individuals was the “absence of eggs”. Because body size and physiological state in *Daphnia* depend on parental history, applying more stringent criteria—such as post-hatch age (neonate age) and body length—could further reduce between-subculture variability in future work. It should also be noted that strict determination of post-hatch age is practically challenging in this system because the individuals swim freely.

### Future perspectives

Several priority research tasks are envisaged for the further development of the DMC constructed in the present study.

The most important is the direct quantification of vitamins and the analysis of microbial communities. By combining HPLC-based quantification of B12, B1, and B7 with 16S rRNA gene analysis or metagenomic analysis, it should be possible both to obtain direct evidence for in-system vitamin biosynthesis within the DMC and to identify the producer organisms. In particular, disentangling the respective contributions of sediment anaerobic bacteria and *Daphnia*-associated bacteria is essential for understanding the vitamin-supply mechanism in the DMC.

In addition to direct quantification, combining it with a manipulative experiment in which B vitamins are supplied exogenously to a system that lacks an anaerobic sediment (for example, the coarse glass beads used in the present study) could functionally test whether vitamin availability is a limiting factor for *Daphnia* growth. This manipulation would not, however, constitute a single-variable control, because changing the sediment simultaneously alters the composition of the microbial and algal community, exogenously added vitamins are subject to uptake and competition by the community, and vitamins supplied directly to the water column are not equivalent—in their timing, localization, and route—to those obtained through the internal cycling of the food web. The more decisive priority is therefore the direct analytical verification consisting of quantifying the vitamins and identifying their site of biosynthesis and their transfer route to *Daphnia*; such manipulative experiments are positioned as a complementary, secondary line of evidence.

To broaden the applicability of the DMC, the establishment of culture conditions capable of maintaining *Daphnia* populations across multiple generations is also an important task. In the present study, *Daphnia* abundance declined after reaching its peak in every subculture, and this may be related to the initial setting of medium elemental concentrations (‘Limitations’, fourth paragraph). In addition to systematic optimization of medium elemental concentrations, semi-open operation with periodic nutrient inputs mimicking rainfall and stream inflow in lakes is also worth considering as an option for long-term maintenance. Whether the introduction of such external inputs is compatible with “reproducibility as a closed system”—a key advantage of the present approach—needs careful consideration. As shown by the review of Padilla Suarez et al. (2023), multigenerational *Daphnia* tests (including those extending to six generations) are gaining importance for assessing the chronic effects, transgenerational responses, and adaptive resistance of chemicals, and the establishment of a multigenerational maintenance culture system in the DMC would form the basis of long-term exposure tests.

Building on the DMC, an extension to microcosms that include fish is also envisioned. Because algae and *Daphnia* already coexist in the DMC, the addition of fish that prey on these would enable observation of the biomagnification of chemicals through the food web. However, adding fish requires structural adaptations such as ensuring refuges for *Daphnia* and scaling up the system.

Ultimately, conducting chemical toxicity tests using the DMC and observing not only *Daphnia* population dynamics but also changes in microbial community structure in the sediment and water column will allow the results to be compared with those of existing microcosms (such as the Kurihara microcosm) and mesocosm tests, providing a quantitative assessment of the utility of the DMC as an ecotoxicological tool. In addition, testing whether the established DMC community can be reconstructed and maintained consistently across independent laboratories following transfer of the community (inter-laboratory reproducibility) will be an important step toward establishing the DMC as a standardized testing platform.

## Conclusions

In this study, an S-type microcosm (DMC) was constructed by combining a DMC medium containing all 31 essential elements as inorganic compounds with a quartz-sand sediment of 0.3–0.6 mm grain size. Across three successive subcultures, *Daphnia* grew consistently from three founders to a mean peak abundance of 23.8 ± 3.2, confirming the reproducibility of the system construction.

The preliminary experiment indicated that the presence of sediment and an appropriate grain size play an important role in the formation of an anaerobic environment and the diversification of the algal community.

These observations are consistent with the working hypothesis that microbial communities in the anaerobic sediment biosynthesize B vitamins (B12, B1, B7) and supply them to *Daphnia* through the food web. However, vitamins were not directly quantified and microbial community composition was not characterized in this study; the in-system biosynthesis hypothesis therefore remains to be tested directly.

The DMC is presented as a complement and extension of existing microcosm methods at the level of system design—comprehensive elemental coverage and a sediment-based anaerobic environment—and is positioned as a platform on which direct analytical verification of in-system nutrient biosynthesis and chemical toxicity testing can be conducted in future work.

## Supplemental Information

10.7717/peerj.21882/supp-1 Supplemental Information 1 *Daphnia* population dynamics across three successive subcultures (Subcultures I–III) of the *Daphnia* Microcosm (DMC)

10.7717/peerj.21882/supp-2 Supplemental Information 2 Representative photomicrographs of biota co-occurring with *Daphnia* in the DMC

10.7717/peerj.21882/supp-3 Supplemental Information 3 Time course of dissolved oxygen (DO) within the glass-bead sediments of two grain sizes during the preliminary experiment

10.7717/peerj.21882/supp-4 Supplemental Information 4 Time course of dissolved oxygen (DO) in the culture solution of the DMC with quartz-sand sediment (0.3–0.6 mm grain size) during the main experiment

10.7717/peerj.21882/supp-5 Supplemental Information 5 Time course of culture-solution temperature in the DMC during the main experiment

10.7717/peerj.21882/supp-6 Supplemental Information 6 Conceptual overview of the DMC Schematic summary of the DMC concept. A purely inorganic medium containing all 31 essential elements with no added vitamins, combined with a fine quartz-sand sediment (0.3–0.6 mm grain size), forms an anaerobic zone within the sediment in which microorganisms are hypothesized to biosynthesize B vitamins (B12, B1, B7) that are supplied to *Daphnia* through the food web. In the output panel, the smooth curve is an illustrative trend rather than a measured time series, whereas the endpoint value (mean peak abundance 23.8 ± 3.2 individuals from three founders, *n* = 3) is an actual result of this study (see Fig. 6 for the measured population dynamics).

10.7717/peerj.21882/supp-7 Supplemental Information 7 Original, unedited photograph of the *Daphnia* specimen shown in Fig. 1 The original micrograph from which Fig. 1 was prepared, provided for full transparency. In Fig. 1 the background of this same photograph was retouched with Gemini (Google) to give the grid and surrounding field a more uniform appearance; the *Daphnia* specimen itself—its body outline, appendages, and internal structures—is identical in both images and was not altered, moved, or morphologically modified. The background grid is a slide glass with 1-mm grid spacing, providing a length reference. The clonal lineage was morphologically similar to the *D. pulex* group and was collected from a pond near Fukushima University. Photo credit: Kunihiko Kakazu.

## Additional Information and Declarations

### Data Availability

The following information was supplied regarding data availability: The data is available in the Supplemental Files.
