# Supplementary material for: Construction of a Daphnia-sustaining microcosm using purely inorganic medium: a system designed to investigate endogenous nutrient biosynthesis
Source: PeerJ. 2026 Oct 29;14:e21882. doi: 10.7717/peerj.21882 (PMC13724051; doi:10.7717/peerj.21882)
Supplement: Supplementary file 1 [file peerj-14-21882-s002.pdf]

Supplementary Information

Construction of a *Daphnia*-sustaining microcosm using purely inorganic medium: a system designed to investigate endogenous nutrient biosynthesis

Kunihiko Kakazu Unaûliated, Naha, Okinawa, Japan

Fig. S1. Representative photomicrographs of biota co-occurring with *Daphnia* in the DMC

Light microscopy of the stabilized DMC revealed bacteria, eukaryotic algae, a heliozoan protozoan, and a small annelid co-occurring under purely inorganic conditions.

Imaging conditions: light microscope, 10× eyepiece with 4×/10×/40×/100× objectives. Total magnification is indicated for each panel. In panel (h), the background grid is a slide-glass grid with 1-mm spacing.

Photo credit: Kunihiko Kakazu.

(a) Community overview — 1000×

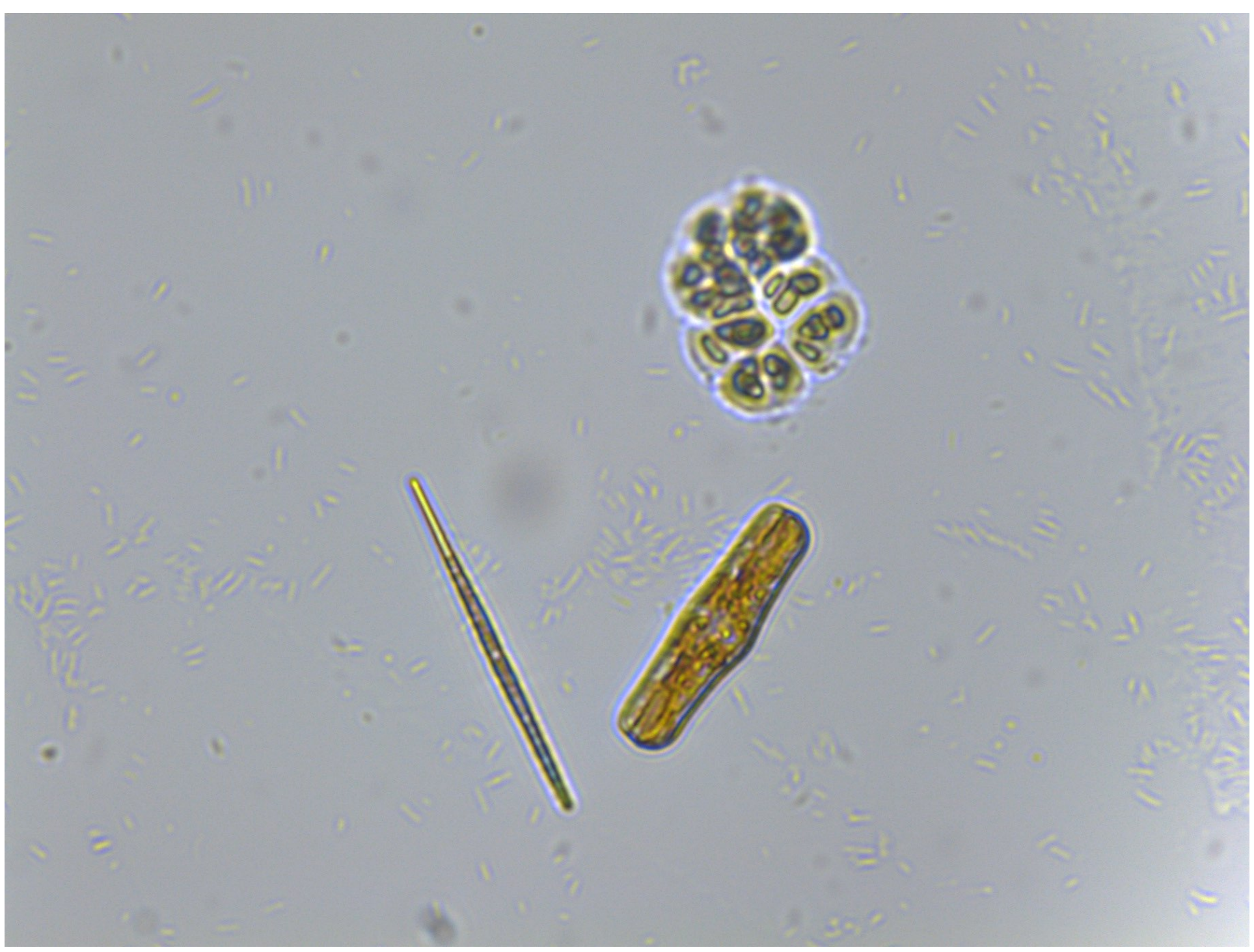

A colonial green alga, two pennate diatoms, and background bacteria in a single field.

(b) Filamentous green alga — 400×

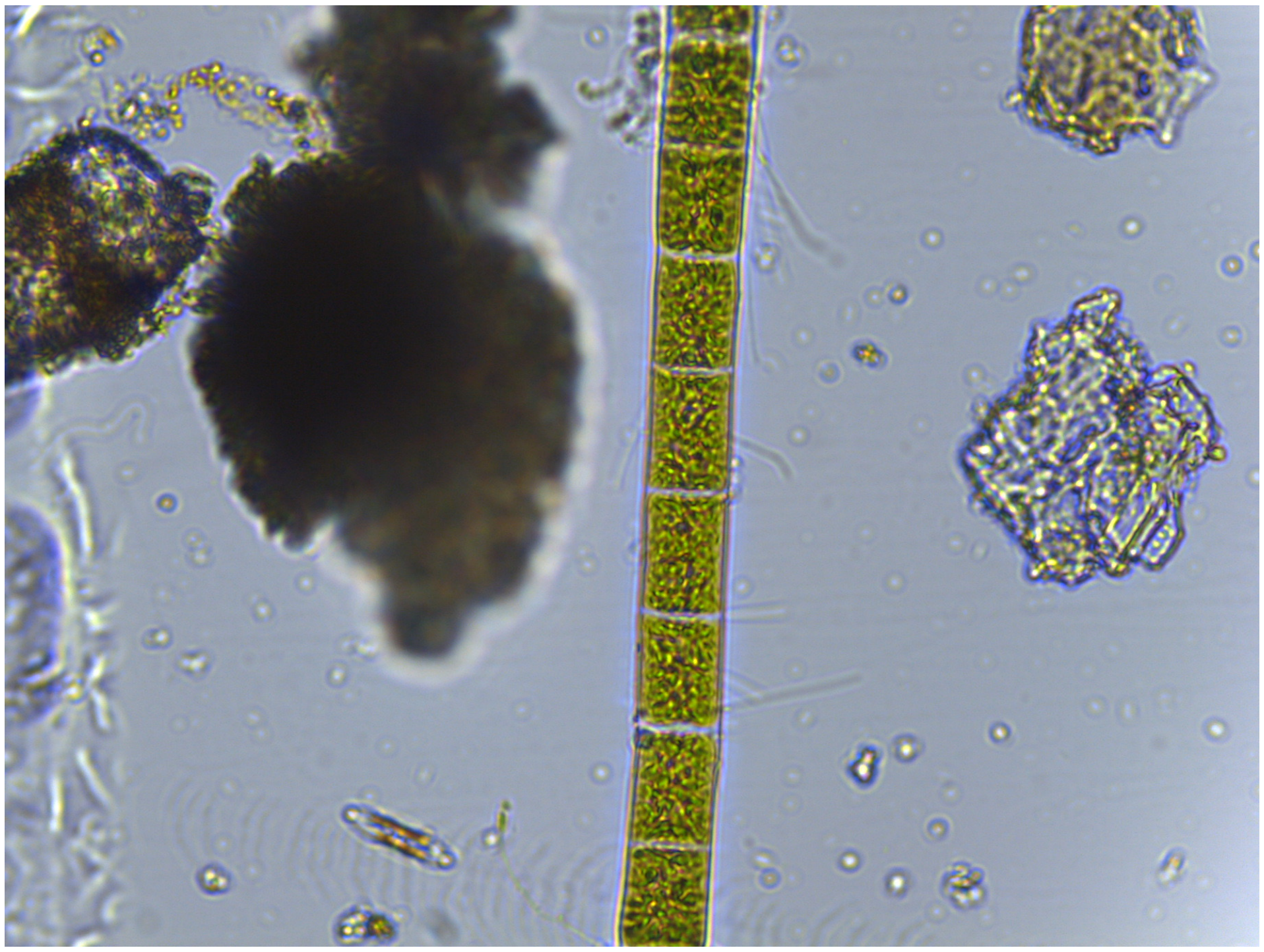

Filamentous green alga with detrital aggregates.

(c) Pennate-diatom aggregate — 1000×

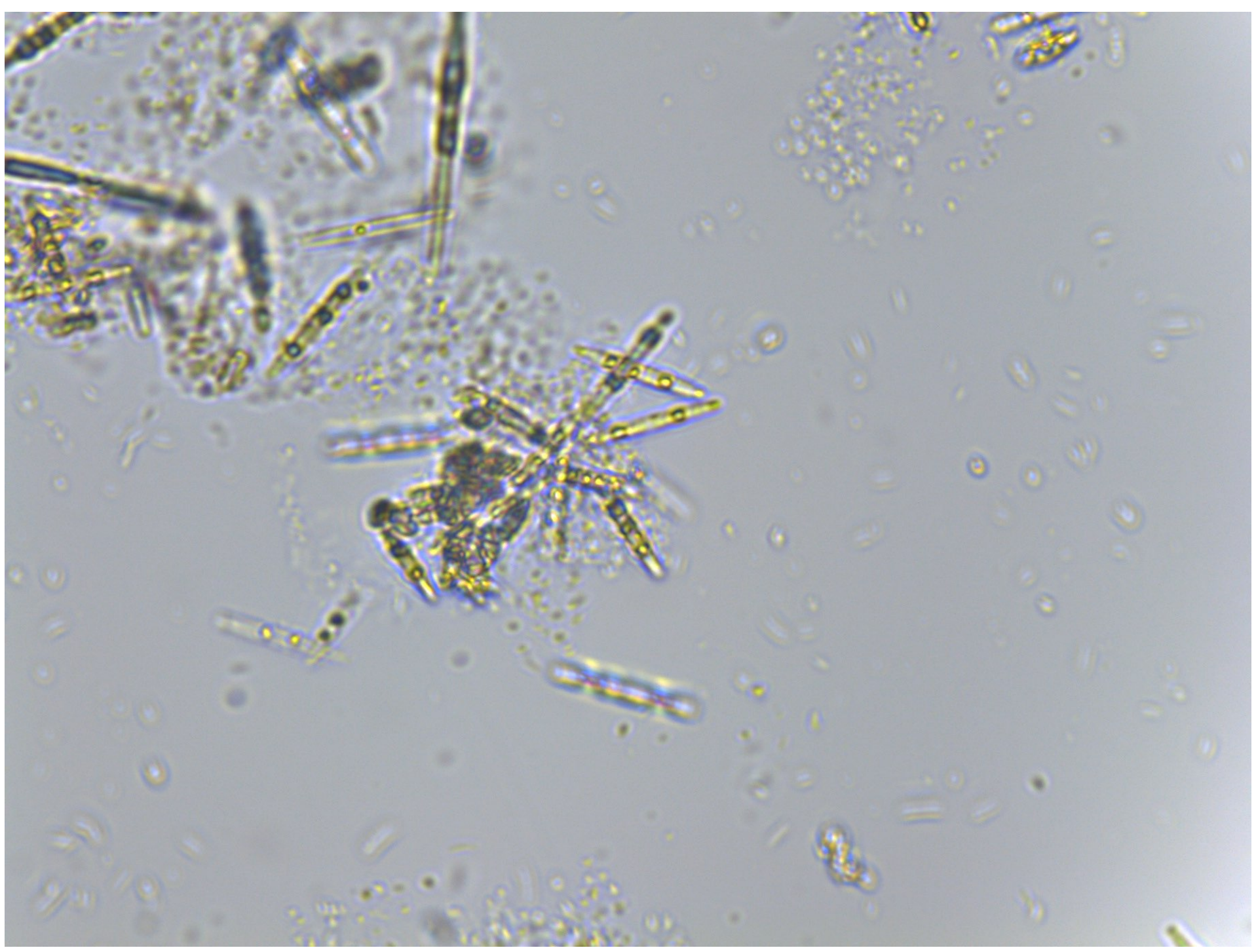

Pennate diatoms forming a star-like cluster around a central particle.

(d) Elongate pennate diatom — 1000×

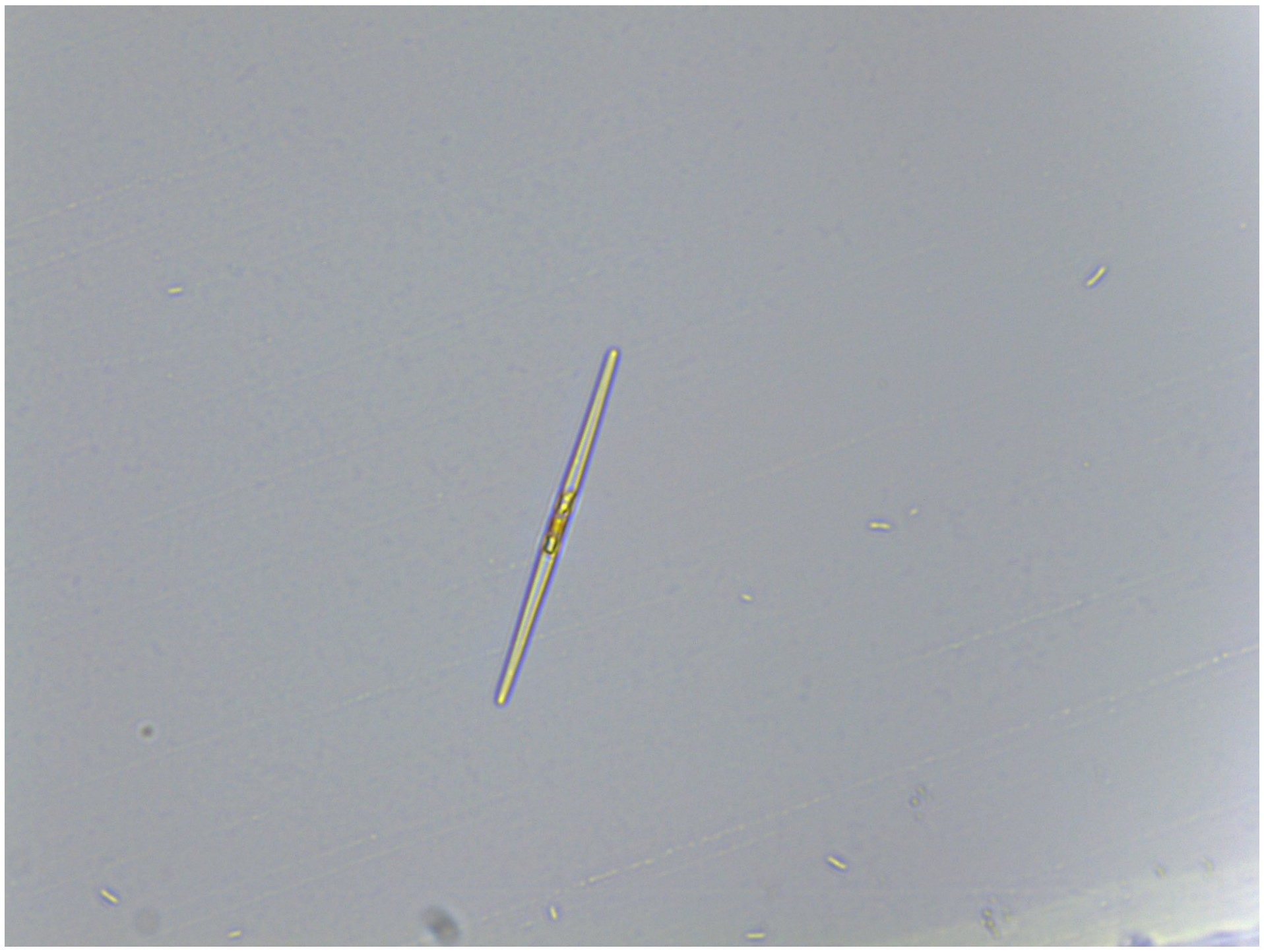

A single needle-like pennate diatom.

(e) Pennate diatoms (thicker morphotype) — 1000×

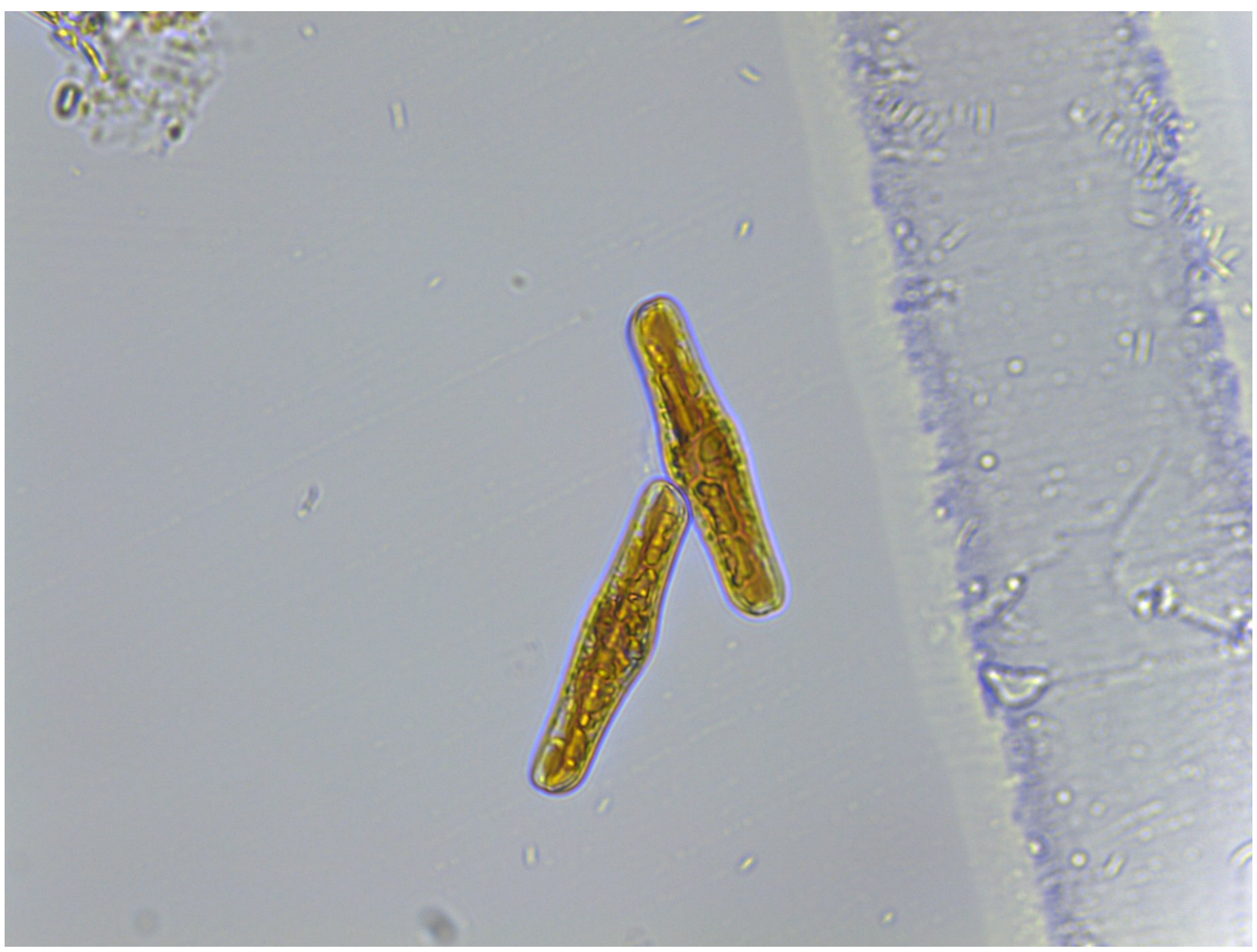

Lanceolate pennate diatoms, distinct from the elongate morphotype in (d).

(f) *Scenedesmus* sp. — 1000×

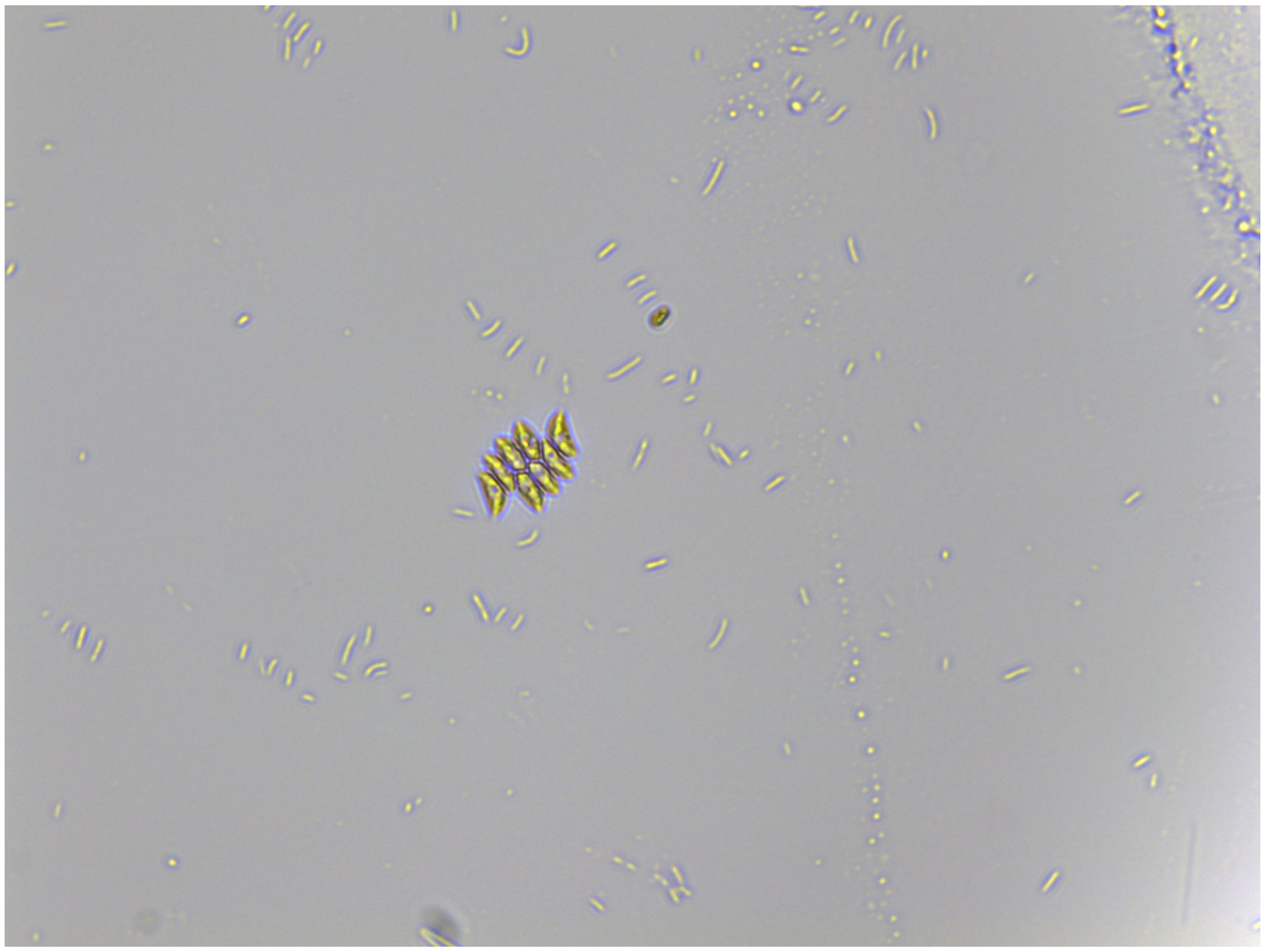

A coenobium of *Scenedesmus* sp. (close to *S. dimorphus*) with background bacteria.

(g) Heliozoan — 1000×

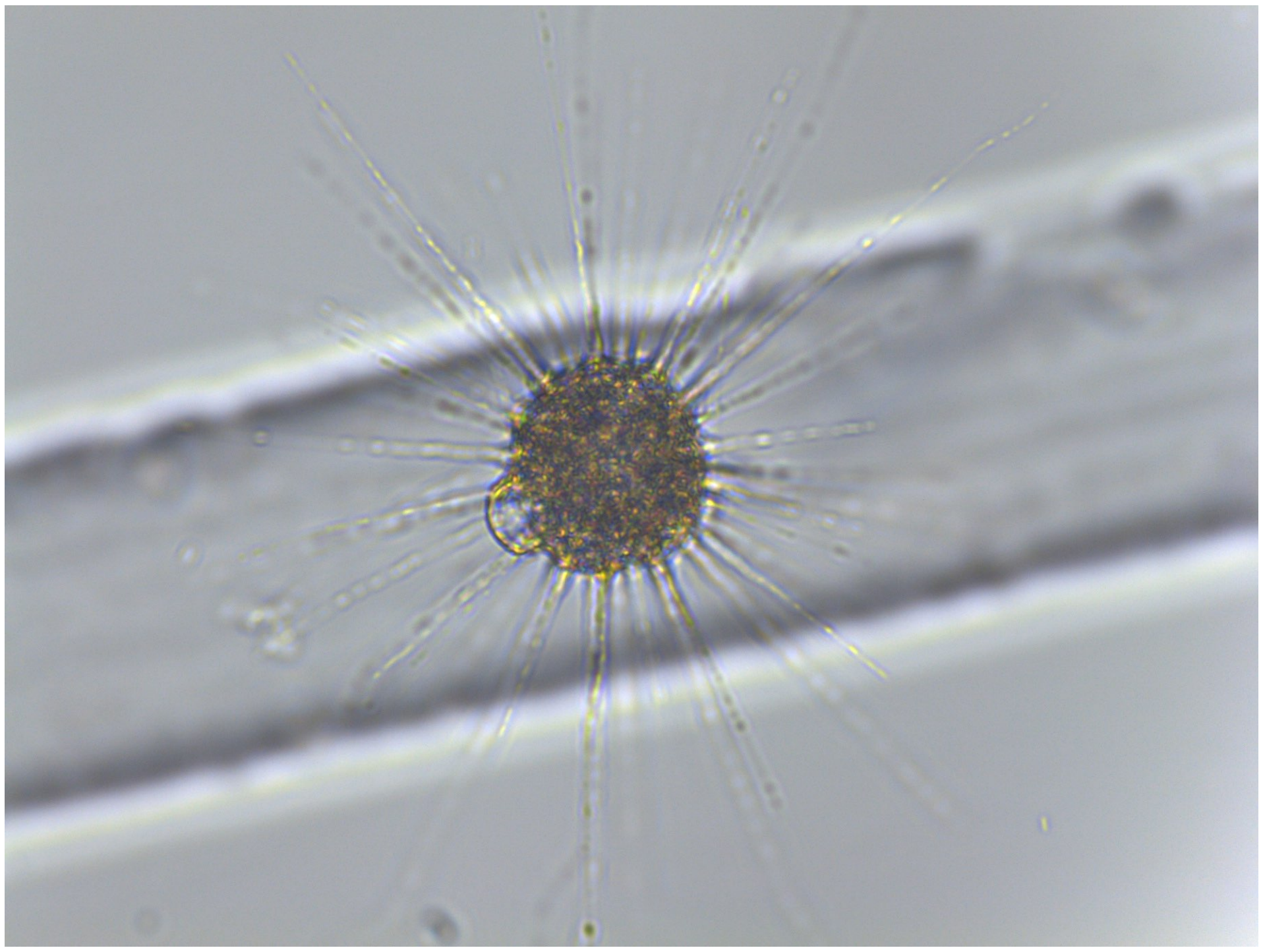

A heliozoan protozoan with radial axopodia.

(h) *Aeolosoma* sp. — 100×

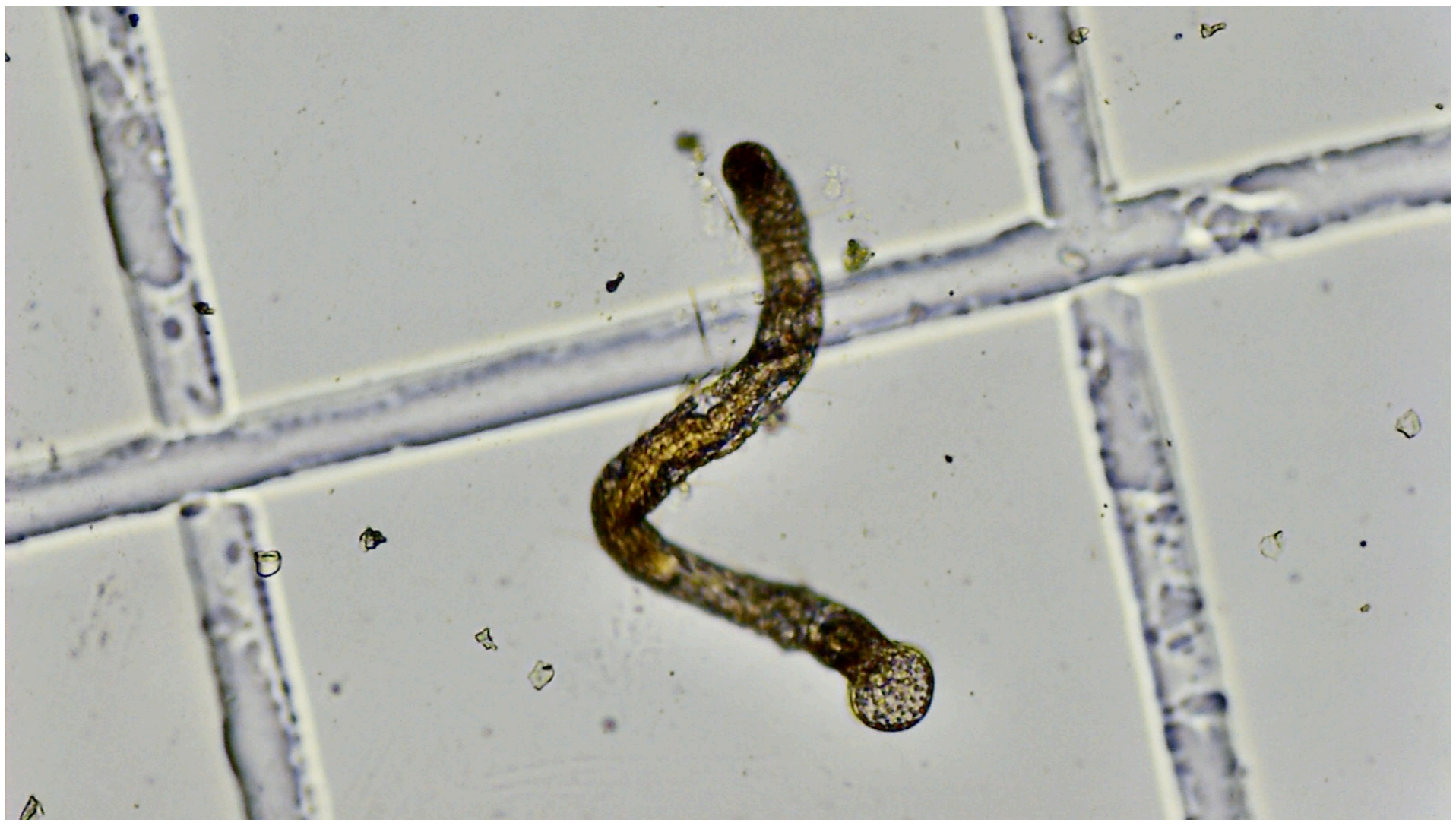

Aeolosoma sp. (Aeolosomatidae); body length ≈ 1 mm against the 1-mm slide-glass grid.
